# Some Points in the Treatment of Diphtheritic Laryngeal Obstruction.—I

**Published:** 1911-07-15

**Authors:** A. Knyvett Gordon

**Affiliations:** formerly Medical Superintendent of Monsall Hospital and Lecturer on Infectious Diseases in the University of Manchester.


### Hospital Clinics.

### SOME POINTS IN THE TREATMENT OF DIPHTHERITIC LARYNGEAL OBSTRUCTION.—I.

By A. KNYVETT GORDON, M.B., Cantab., formerly Medical Superintendent of Monsall Hospital and Lecturer on Infectious Diseases in the University of Manchester.

When diphtheria attacks the larynx we almost invariably get some degree of respiratory obstruction, which may vary from merely an interference with the sound of inspiration—the patient meanwhile obtaining sufficient air for his requirements—to death from suffocation. It not infrequently happens that the successive stages of this invasion succeed one another with almost dramatic rapidity, so that the medical attendant may be confronted with a condition which obviously requires immediate and active treatment at a time and place which may tax his resources very considerably.

For some reason, the text-books which are most frequently read by the average student are not as clear as they might be on the treatment of laryngeal obstruction in diphtheria, and some of them undoubtedly tend to produce in the mind of the neophyte the impression that the necessary surgical procedures are fraught with terrorising difficulties. I am sure, from my own experience, that there are many men who in an emergency would open an abdomen with confidence but would "funk" a tracheotomy. This is partly due to a want of clearness as to the meaning of the signs of laryngeal obstruction, and partly to some inherent defects in the operation for their relief as it is usually described. As it has fallen to my lot to watch the perturbations of not a few residents in the performance of their first tracheotomy, I shall deal with the subject mainly from the hospital point of view, though, as I shall have occasion to point out, the impression—that undoubtedly prevails—that the necessary procedures are almost impossible in the poorer class of private practice is quite erroneous.

We will deal with the symptoms in the order of their occurrence, and it will be found that they naturally divide themselves, both on pathological and clinical grounds, into four stages, and we can conveniently describe two degrees in each stage. They are as follows:—

Firstly, the stage of stridor—that is to say, the act of respiration instead of being almost inaudible becomes noisy; at first the noise is only apparent when the patient coughs, but later, there is some stridor mainly with inspiration, but, if we listen carefully, we shall find that expiration also is more apparent than it should be. Not only are the cough and inspiration louder than normal, but the pitch is raised so that we get a sound to which the name of croup has been given. This name is hardly a good one, as it has been applied to so many different conditions; but it should really be reserved for the sound only and not applied to any disease with which that sound is associated. So our two degrees are—firstly, croupy cough and then croupy breathing. While on this point let me add a word of caution against confusing laryngeal with pharyngeal stridor; the two sounds are really quite different, but I have many times seen patients sent into hospital for immediate tracheotomy when the stridor was of tonsillar origin only, and surgical interference was not at all necessary. This stage of laryngeal stridor is present in practically all cases of diphtheria where the larynx is at all affected, and by itself it has no dangers.

The next stage is that of muscular effort.

Here the patient is getting enough air for his requirements, but with difficulty, and he has to use for the purpose muscles which are not usually employed during normal respiration. In the first degree of this stage we see the alae nasi working, and the sterno mastoids in action; the head is fixed on the pillow, and the thorax is pulled up with each inspiration. In the second degree we have also retraction, or recession at the epigastrium and at the root of the neck with each inspiration. This sign, however, is often misinterpreted, and it is important that we should remember the age of the patient in estimating its significance. In babies, retraction may occur when there is no laryngeal obstruction whatever; we see it frequently when they are suffering from broncho pneumonia and even when they cry or struggle, while an older child may have considerable obstruction in its larynx with little or no retraction. I remember well the only case of diphtheritic laryngeal obstruction in an adult that I have ever seen, and there, though a post-mortem examination revealed an almost complete occlusion of the larynx, there was no epigastric recession whatever. In young children, I have often seen tracheotomy performed quite unnecessarily merely because this sign was present in a marked degree. In a child of five years old or thereabouts it should impel us to watch, but not necessarily to operate

The next stage is that of deficient aeration.

Hitherto respiration has been adequate in the sense that, though much effort was required, the blood has received its proper supply of oxygen; but as the obstruction increases, the blood becomes more venous in character, so that it is no longer able to convey a sufficient quantity of oxygen to the tissues.

The first degree in this stage is denoted by the restlessness of the patient; he is constantly changing his position, and may often be seen trying to climb up the sides of his cot, or standing up in bed; this restlessness increases, and very soon the next degree—that of cyanosis—is reached. This shows itself first in a duskiness of the lips and of the tips of the ears, and it is without doubt the most important sign we have to observe in diphtheritic laryngeal obstruction.

The next stage is that of asphyxia, and the point to note here is that the signs of effort, of discomfort, and of distress all pass off; the child lies down in bed, the stridor diminishes, and the child apparently sleeps, but it is euthanasia, from which the patient passes quite gradually into the second degree, death from suffocation.

### When to Interfere.

We have now to decide firstly when we shall interfere, and then what we shall do. Before interfering we may take it for granted that we should give antitoxin directly we come to the conclusion that we have to deal with a case that may even possibly be one of diphtheria, unless the patient has had a dose previously, in which case we may wait a little while, bearing in mind the danger of the phenomena due to anaphylaxis or supersensitation. If, however, the obstruction is well marked, or the degree of toxæmia intense, we must give antitoxin, and risk the development later of serum symptoms.

The question then arises, how long can we afford to wait before operating (and after administration of antitoxin) in the hope that surgical interference may not be necessary? Obviously, the most important factor here is the date of origin of the attack. On the first day of disease, the use of antitoxin practically always renders subsequent operation unnecessary, and if given on the second day, the chances of improvement without interference are very good; but after the third day I believe personally that antitoxin is almost useless, and in any given case, we ought not to wait at this stage in the hope that the obstruction will diminish. In common with most other observers, I have given enormous doses of serum at this stage in the endeavour to compensate for the omission to give a normal dose at an earlier stage, but I am not convinced that they have ever been successful.

During the first three days then—assuming that the dose of serum has been accurately gauged, a point to which I will return later—we may expect some signs of its action to show themselves in from three to six hours after administration. We have, in any given case, to decide whether we can safely leave the patient for this time.

### Indications for Surgical Interference.

What, then, are the indications for surgical interference? Here we must make a distinction between cases in hospital where the surgeon is on the premises (and it must be remarked in passing that a resident medical officer is essential for the safety of the patients in a hospital to which cases of diphtheria are admitted), and those occurring in private practice, especially in the country, when the medical attendant cannot always be found at once. In hospital cases, provided that the resistance of the patient, as estimated by the rate and character of the pulse, is fairly good, we can safely postpone operation for three or four hours even if the patient is restless, provided that there is no cyanosis. If the lips are blue we must always operate at once.

If, however, the pulse is rapid, it is best to interfere when the patient is restless. In private practice, unless the surgeon can attend to a summons immediately, it is advisable to operate if the retraction is well marked without waiting for restlessness. In any case, if the patient is having attacks of acute obstruction with quiet intervals, the probability is that there is a piece of loose membrane below the larynx, and we should always open the windpipe in a quiet interval. Non-interference in this class of case has been responsible for many of the tragedies connected with diphtheria.

### Dosage of Antitoxin.

Before discussing the nature of the operation to be performed, we must return to the question of the dosage of antitoxin. Here there are many different opinions, and I can only give that to which my own experience has led me. On the first day of disease, I believe a dose of 4,000 units to be sufficient for any type of case, and in mild cases, 2,000 units is usually adequate. On the second and third day we should distinguish a little and give 6,000 units if there is no membrane to be seen on the fauces—whatever the degree of laryngeal obstruction—and from 8,000 to 10,000 units if the fauces are also affected. In patients who are intensely toxæmic, I have often found that 4,000 units given intravenously has acted like a charm. Though I was accustomed at one time to give doses of 20,000 units to all bad cases almost as a routine practice, I do not now think that we need go beyond 10,000 units as a rule. This is not because I have observed any bad effects from the larger doses—for the degree and nature of serum symptoms do not appear to be related to the quantity administered—but because the extra 10,000 units seem to be unnecessary. The age of the patient need not, in practice, be considered in relation to the dose, probably because the younger the child the less the resistance to the diphtheritic toxin, and so a proportionally larger dose of antitoxin is required.

*(To be continued.)*

